# Post-Exposure Prophylaxis and Methamphetamine Use among Young Sexual Minority Men: The P18 Cohort Study

**DOI:** 10.3390/ijerph19020712

**Published:** 2022-01-09

**Authors:** Elizabeth Kaplun, Richard J. Martino, Kristen D. Krause, Michael Briganti, Paul A. D’Avanzo, Perry N. Halkitis

**Affiliations:** 1 Center for Health, Identity, Behavior, and Prevention Studies, Rutgers School of Public Health, Newark, NJ 07102, USA; lizkaplun@gmail.com (E.K.); richard.martino@utas.edu.au (R.J.M.); kristen.krause@rutgers.edu (K.D.K.); mb1629@sph.rutgers.edu (M.B.); pad196@newark.rutgers.edu (P.A.D.); 2 Department of Epidemiology & Biostatistics, Rutgers School of Public Health, Piscataway, NJ 08854, USA; 3 Department of Urban-Global Public Health, Rutgers School of Public Health, Newark, NJ 07102, USA; 4 Department of Health Behavior, Society, and Policy, Rutgers School of Public Health, Piscataway, NJ 08854, USA

**Keywords:** substance use, sexual health, sexual minority, gender minority, racial and ethnic minority, syndemic theory

## Abstract

Methamphetamine use is associated with increased risk of HIV infection among young sexual minority men (SMM). Post-exposure prophylaxis (PEP) is an effective strategy for individuals who are exposed to HIV, but there is limited research about PEP use among young SMM and its relationship with methamphetamine use. This study analyzes the association between ever PEP use and recent methamphetamine use among young SMM in New York City, using cross-sectional data from the P18 Cohort Study (*n* = 429). Multivariable logistic regression models were used to assess the association between methamphetamine use and ever PEP use. Compared with those who had not used methamphetamine in the last 6 months, young SMM who did use methamphetamine were significantly more likely to have ever used PEP (AOR = 6.07, 95% CI: 2.10–16.86). Young SMM who had ever used PrEP had 16 times higher odds of ever using PEP (AOR = 16, 95% CI: 7.41–35.95). Those who completed bachelor’s degrees were 61% less likely to have ever used PEP (AOR = 0.39, 95% CI: 0.17–0.88). These data suggest that methamphetamine use could increase the risk of HIV infection, highlighting the critical need to target interventions for young SMM who use methamphetamine and are more likely to engage in unprotected intercourse.

## 1. Introduction

Rates of HIV infection have declined significantly over the last decade, but disparities persist among vulnerable populations. The Center for Disease Control and Prevention (CDC) reports that approximately 36,400 people were newly infected with HIV in the United States in 2018 [1]. Of these new diagnoses, 69% were men who have sex with men (MSM) or sexual minority men (SMM), with young SMM aged 13–24 compromising 80% of new diagnoses in their age group, and 25% of all SMM [2]. Additionally, racial and ethnic minority men are disproportionately affected by HIV.

Among young SMM diagnosed with HIV in 2018 (ages 13–24), 47% were non-Hispanic (NH) Black, 31% were Hispanic/Latino, and 15% were NH White [2]. 

Although there has been much progress since the height of the AIDS epidemic in the mid-1980s, progress has stalled in recent years, with nearly constant annual incidence rates since 2014 [1]. In the last few decades, HIV has become a chronic condition that is highly manageable through antiretroviral therapy [3], and in 2012, the U.S. Food and Drug Administration approved Truvada^®^, the first pre-exposure prophylaxis (PrEP) medication for people who are HIV negative but are at high risk of contracting HIV [4]. This was a monumental medical achievement for HIV prevention, and has proven to be highly effective when used appropriately [4]. Recent studies, however, suggest that while many have heard of PrEP, uptake among SMM has been low, only about 3.4% ever using it, particularly among racial and ethnic minorities, who are at higher risk of HIV infection [2,5,6]. 

On the other hand, post-exposure prophylaxis (PEP) is a drug treatment administered once daily for 28 days, meant to prevent HIV infection after exposure [7]. It is also highly effective in preventing HIV, but it is not as well-known as PrEP, and is generally even more underutilized. Current literature about PEP uptake is limited, but one study examining PEP use reported that 7.4% of participants in a New York City, NY (NYC) sample composed of young SMM, transgender women, and cisgender women of color, had ever used PEP, and only 13.2% were aware of PEP [8]. If taken for 28 days and initiated within 72 hours of exposure to HIV, PEP can reduce the odds of infection by 81% [9]. This underutilization of PEP can be partially explained by barriers such as lack of awareness, stigma, and access among high-risk populations [8]. 

In recent years, individuals who engage in sexualized drug use, or “chemsex,” behaviors have emerged as a potentially vulnerable group at risk for HIV infection [10]. In particular, methamphetamine use has been associated with high-risk sexual behaviors and HIV infection among SMM [11]. Due to these associations in emerging young SMM, it is crucial to understand current PEP uptake in this population to better implement and cater education and outreach for HIV prevention for high-risk populations.

Methamphetamine is a strong and addictive central nervous system stimulant that can elevate one’s energy, alertness, concentration, and mood; however, it is also associated with depression, anxiety, psychosis, violent behavior, and overall poor health outcomes [12]. It is also referred to as crystal meth, chalk, ice, and can be orally administered, smoked, injected, snorted, and “booty bumped” (administered rectally) [12,13]. Methamphetamine use has been associated with significant public health problems in the U.S. for over 35 years, with little consensus over how large of an issue it really is. A literature review conducted by Gonzales et al. asserted that national-based surveys depict methamphetamine use as a minor concern, but do not capture the true extent of the issue, whereas data sources from law-enforcement groups, welfare agencies, and substance abuse treatment programs indicate a much more significant problem [11]. 

For sexual minority individuals, rates of methamphetamine use have decreased for those aged 18–25 from 2017 to 2018 (−0.7%), but there has been a resurgence for those aged 26 and older (+1.4%) [14]. These national statistics are important to consider, but they do not shed light on the differences among various regions in the U.S. For instance, a study conducted in NYC showed that while methamphetamine use declined significantly among SMM throughout the 2000s after public health initiatives aimed to reduce methamphetamine use in this subgroup [15], there has been a resurgence since 2011, particularly among SMM communities of color [16]. Another study examined the patterns of methamphetamine use among young MSM in Chicago, Illinois, and found that 13% of participants (*n* = 310) aged 16–24 reported methamphetamine use [17].

These current trends of methamphetamine use are concerning for young SMM emerging into adulthood, who may struggle with psychological distress, self-esteem, and perceived stigma associated with their sexual identity. Research suggests a link between these issues and young SMM partnering with older men in adult social settings, where substance use and casual sexual encounters are more common [17]. Unprotected sex and sexual risk-taking behaviors increase the risk of HIV transmission and infection among SMM. Methamphetamine use is strongly associated with sexual risk-taking as well, and research shows that this stimulant elevates levels of impulsivity, desires to engage in unprotected sex, and sex with more than one and/or anonymous partners [18,19].

Overall, the emerging literature about methamphetamine use in SMM populations and the high risk of HIV infection indicates a growing need for more research and the implementation of science to better focus primary and secondary prevention efforts. Although PrEP is a highly effective preventative measure young SMM can take to significantly lower their risk of HIV infection, uptake is reported to be quite low. In a NYC survey, 41% of SMM had heard of PrEP, but only 3.4% of the sample had ever taken it [5]; in general, PrEP uptake rates are lower among young SMM than older SMM [20]. Among SMM, researchers found that PrEP awareness was significantly lower for methamphetamine users when compared with those who do not use methamphetamines (89% and 95%, respectively, *p* = 0.01), but there was no significant difference in actual PrEP use between the two groups [21]. 

The current literature about PEP and methamphetamine use in the young SMM population is extremely limited. One retrospective cohort study sought to evaluate any association between PEP prescriptions at a community health center in Boston, MA and methamphetamine use during the HIV exposure event and chronic methamphetamine use. This study found that frequent methamphetamine users came back for repeat PEP prescriptions more than non-methamphetamine users (AOR = 5.13, 95% CI: 2.82–9.34) but were significantly more likely to seroconvert during follow-up periods (AHR = 3.61). Additionally, methamphetamine use was associated with increased odds of unprotected anal intercourse (AOR = 2.12, 95% CI: 1.16–3.87) [22].

It has also been documented that healthcare providers may be hesitant to prescribe PEP to stimulant users, due to observations of poor compliance with the therapy among methamphetamine users, with significantly lower rates of PEP course completion when compared with non-substance users [23,24]. The public health implications of these medical decisions could be detrimental to young SMM who use methamphetamine, as they are more likely to engage in sexual risk-taking behavior and unprotected sex [25].

Due to the aforementioned gaps in the literature, this study seeks to understand individual level factors associated with young SMM who have ever used PEP. The aim of this study was to evaluate the association of ever PEP use and methamphetamine use (within the last 6 months) among young YSMM living in the New York City metropolitan area (NYC). It was hypothesized that there was a significant association between those who reported using methamphetamines in the past 6 months (at time of follow-up) and using PEP at least once ever.

## 2. Materials and Methods

### 2.1. Study Design and Data Sources

The data for this cross-sectional analysis were drawn from the baseline of the second wave of the P18 Cohort Study, a prospective cohort study that surveyed young sexual minority men (YSMM) in NYC to assess the syndemic of substance use, mental health burden and HIV. Study details were previously published [26] and are briefly summarized here. Briefly, recruitment for the study was conducted in two waves, Wave I (2009–2014) recruited a total of 600 young SSM between the ages of 18–19, and Wave II (2014–2019) included participants from the original cohort and new age-matched participants for a total of *n* = 665 participants between the ages of 22 and 23. Participants were recruited through a number of active and passive methods, including in-person events (e.g., PRIDE festivals, community meetings), online methods (e.g., mobile applications and websites), and referrals from other participants. At each study visit (every six months), audio computer-assisted self-interviews (ACASI) were used for participants to provide details on sociodemographic factors, psychosocial factors, and drug use. Participants also completed a timeline follow back (TLFB), a calendar-based, semi-structured interview used to evaluate event-level behavioral data for the 30-day period before the assessment to assess sexual behavior [27]. Study participants were also tested for HIV serostatus with a rapid finger prick antibody test. Finally, participants were compensated at each study visit.

### 2.2. Study Participants

Young sexual and gender minority young adults who were assigned male at birth (*n* = 665) were recruited for the P18 Cohort Study. Participants were eligible for the study if their HIV status was negative or unknown at the time of screening, if they were age 22–23, and assigned male at birth. Participants also must have reported having sex with a man in the last 6 months at the time of screening.

### 2.3. PrEP and PEP Use

Ever PrEP use was determined through the question “have you ever taken PrEP?” Participants were provided with the definition of PrEP as “an HIV-negative person taking a daily pill to prevent HIV” when answering this question. Ever PEP use was determined through the question “have you ever taken PEP?” 

### 2.4. Methamphetamine Use

To determine ever methamphetamine use in the last 6 months, all forms of use were categorized into one variable. The questions that were combined were: “In the past 6 months, have you injected crystal, ice or tina (methamphetamine)?”; “In the past 6 months, have you smoked/snorted crystal (meth)?”; “In the past 6 months, have you booty bumped crystal (meth)?” These questions were used to create a binary variable if a participant had used methamphetamines through any route of administration in the last 6 months (at time of follow-up).

### 2.5. Unprotected Sexual Behavior

Participants were asked about various unprotected sexual behavior, capturing the number of given and/or received unprotected sexual acts the participants performed in the last month using the TLFB. These behaviors were: total unprotected oral acts given (past month), total unprotected oral acts received (past month), total unprotected anal acts given (past month), total unprotected anal acts receptive (past month), total unprotected mutual masturbation acts (past month), total unprotected rimming acts given (past month), total unprotected rimming acts received (past month), and total unique partners (past month).

### 2.6. Sociodemographic Characteristics

All sociodemographic data was self-reported by the participant. There were six racial/ethnic groups in this study consisting of Hispanic/Latino, Non-Hispanic (NH) Black, NH Asian, NH Mixed, NH Native American/Other, and NH White. Nation of birth was dichotomized into US-born and foreign-born. Sexual identity was determined by using the Kinsey scale by asking participants “Do you identify as homosexual?” with responses ranging from identifying as not exclusively homosexual (0) to exclusively homosexual (6) [28]. These responses were dichotomized for this study as exclusively homosexual versus not exclusively homosexual. Total income was self-reported by participants with a 12-category response set, but was then recoded into “Low” (<$5000), “Middle” ($5000–19,999), and “Upper” (≥$20,000) income categories, to match a previously published study using the same dataset [29]. Education status was categorized into four groups of educational attainment: high school completion or lower, associate degree, bachelor’s degree, and graduate degree. Finally, participants were asked about their HIV status, and their responses were confirmed through rapid antibody testing by finger prick. Participants from Wave I who tested positive for HIV had previously received viral load testing, as well.

### 2.7. Analytic Plan

The software R and RStudio (version 3.6.1) were used for three levels of data analysis. First, descriptive statistics were generated for all measures. Second, chi-squared bivariate analyses were performed on the frequency of sociodemographic factors, PrEP use, methamphetamine use in the last 6 months by PEP use, and risky sexual behaviors. Statistical significance was set at two-tailed α < 0.05. For some analyses, Fisher’s Test was used in place of chi-squared testing, due to expected cell sizes under 5. Measures that were statistically significant in the bivariate tests were included in the full multivariable logistic regression model. Third, the association between using methamphetamine in the past 6 months as the main exposure variable and ever using PEP as the main outcome variable was determined through univariate logistic regression modeling in the main unadjusted model; univariate logistic regression models for demographic and behavior measures were also used to assess association with PEP use individually. Finally, an adjusted multivariate logistic regression model was created for ever use of PEP (reference = no) as the dependent variable and ever using methamphetamine in the past 6 months as the main independent variable. The final model controlled for race/ethnicity because of priori knowledge [1], as well as educational attainment and PrEP ever use because of statistical significance in the bivariate analyses.

## 3. Results

### 3.1. Sample Characteristics

Table 1 shows the characteristics of the baseline visit for all participants for the second wave of the P18 cohort, conducted at 42 months after the beginning of the first wave. The baseline sample was racially and ethnically diverse, with 31.88% of the participants identifying as Hispanic/Latino, 25.56% identifying as non-Hispanic Black, 25.26% identifying as non-Hispanic White, 7.37% identifying as non-Hispanic Asian, 6.47% identifying as non-Hispanic multiracial, and 3.46% identifying as non-Hispanic Native American or “other”. Most participants were born in the United States (84.21%), and identified their sexual orientation as exclusively homosexual (82.26%). For annual personal income, 32.93% of participants reported a low annual income, 30.08% reported a middle annual income, and 32.18% reported an upper annual income. Only 33.4% of participants were currently enrolled in school at the time of the visit, and 47.8% reported having a high school diploma or less. Participants were mostly HIV negative (95.59%). Only 6.47% of participants reported using methamphetamine in the last 6 months; 8.87% reported using PrEP ever; and 8.57% reported using PEP ever.

### 3.2. Bivariate Analysis

The bivariate relationships between our variables are reported in Table 2, showing frequencies and percentages of PEP status by individual characteristics. Of 665 participants in the Wave II baseline group, there were 236 participants that were removed from the analytic sample because of missing values, most from the PEP and PrEP questions. Our final sample included 429 participants who reported whether they had ever taken PEP. There were no statistically significant differences in PEP use based on race/ethnicity, nation of birth, exclusive homosexual identity, income, school enrollment, or HIV status. There were differences in PEP use by education level, with those who completed a bachelor’s degree having significantly lower frequencies of ever having taken PEP (*p* = 0.03). Ever having taken PrEP was significantly associated with ever having taken PEP (*p* < 0.0001). Finally, reporting having taken any form of methamphetamine in the last 6 months (at time of follow-up) was significantly associated with ever having taken PEP (*p* = 0.002).

T-tests were run for high-risk sexual behaviors and PEP ever use, but there were no significant associations present, and therefore they were not included in the multivariable analysis (Appendix A). 

### 3.3. Multivariable Analysis

Crude models examining ever PEP use by individual characteristics, ever PrEP use, and recent methamphetamine use were fit to determine which variables were significantly associated with the outcome variable. In the univariate analyses, only race/ethnicity, educational attainment, PrEP ever use, and methamphetamine use (in the last 6 months) were significant at α = 0.05, shown in Table 3. These same measures, however, were included in the multivariate logistic regression model because of a priori knowledge or bivariate analysis significance (Table 2).

While not significant in the bivariate model, race was a significant factor in the crude regression model. Specifically, non-Hispanic Black identity was significantly associated with greater odds of ever using PEP (OR = 2.58, 95% CI: 1.17–5.93); no race or ethnicity, however, had significant associations to PEP use in the adjusted model. Those who had ever used PrEP were 16 times more likely to have ever used PEP (95% CI: 7.41–35.95, *p* < 0.0001). Additionally, participants who reported using methamphetamines in the last 6 months (at time of follow-up) had 6.07 times the odds of ever using PEP (95% CI: 2.10–16.86, *p* = 0.0006). Conversely, those who completed bachelor’s degrees were 61% less likely to have ever used PEP than those with just a high school education (AOR = 0.39, 95% CI: 0.17–0.88). 

## 4. Discussion

### 4.1. Methamphetamine Use

This study suggests that recent methamphetamine use is significantly associated with ever using PEP among young SMM in the NYC area, when compared with those who had not recently used methamphetamines. As one of the first studies to assess this association, these findings shed light on young methamphetamine users that are more likely to use a proven HIV prevention method. Stimulant use has been associated with unprotected intercourse and intercourse with HIV positive individuals, particularly among young SMM across many studies [17,18,25], which makes them a vulnerable population in need of resources such as PEP to prevent seroconversion. This study’s findings contribute to this research, suggesting that methamphetamine use is an integral part of the interaction between high-risk sexual behavior and exposure to HIV among young SMM, as suggested by the literature. These findings, however, positively suggest that PEP is being utilized in the methamphetamine-using community to mitigate the risk of contracting HIV.

### 4.2. PrEP Use

The findings also revealed that participants who self-reported ever using PrEP had 16 times greater odds for ever using PEP (*p* < 0.001). This finding suggests that participants who have used PrEP have knowledge of and access to PEP, which is a positive outcome from outreach and prevention efforts dedicated to young SMM. However, more research needs to be done to understand if either form of biomedical HIV protection is predictive of using the other.

### 4.3. Education

Another interesting finding from this study shows that participants who reported having a bachelor’s degree as their highest level of educational attainment reported significantly less ever use of PEP when compared with participants with a high school diploma or less as their highest level of educational attainment (Table 3). There is currently no evidence that educational attainment is associated with PEP uptake, and it is unclear whether this relationship is because those with bachelor’s degrees have less awareness around PEP use or because they are more likely to practice safer sex, and therefore do not seek PEP treatment. More research should be conducted to further understand the individual level characteristics regarding educational attainment as a predictor for PEP use.

### 4.4. Race and Ethnicity

While non-Hispanic Black participants were the only racial/ethnic category more likely to use PEP in a crude logistic regression model, this relationship was not significant after adjusting for methamphetamine use, educational attainment, and PrEP use. Black SMM, however, are disproportionately affected by HIV, and methamphetamine use is a risk factor of interest in understanding sexual risk-taking behavior, although the relationship remains unclear in black populations [30,31]. Jerome and Halkitis (2009) noted that there are strong associations between stigmatization, stress, and substance use within the black SMM community, which can stem from perceived pressure from family members or key figures to present as cisgender and/or heterosexual from a young age [31]. Later on in life, this stigmatization can lead to recovering a sense of belonging and family in the SMM drug-using community [31]. While the association between non-Hispanic Black participants and PEP use in this study does not maintain significance after adjusting for confounders, it is still very important to note that there is a strong initial association, because of a priori literature confirming a strong association to methamphetamine use and HIV seroconversion in the Black SMM community. There is currently no prior literature examining the relationship between race/ethnicity and PEP use, and more research should be conducted to evaluate the disparities in HIV prevention treatments among racial and ethnic groups.

### 4.5. Strengths and Limitations

This study revealed emerging associations that are consistent with the current literature about methamphetamine use and HIV prophylaxis use among young SMM. Additionally, this study sheds light on current trends in a specific sample in NYC which can, in turn, provide direction for public health campaigns and HIV prevention efforts. This study also had several limitations. First, the data was self-reported and did not use biomarker testing to measure recent methamphetamine use, which could allow for recall bias in the participants responses. Although the P18 Cohort Study used longitudinal collection that evaluated responses every 6 months, this analysis was cross-sectional in nature, using only one set of data collected at the start of Wave II, meaning that we cannot determine causality. The dataset was also rather small; 236 respondents with missing data were removed from the data for a total sample size of *n* = 429. Additionally, because participants were comprised of a very limited group of people—those who were aged 22–23 at the time—the significance of factors such as income and educational attainment were limited. As the sample was so young, many of them did not have a substantial yearly income to report, which makes it difficult to examine the role of socio-economic status. Although the scope of the study sought to understand emerging patterns in methamphetamine and PEP use among *young* SMM as they enter adulthood, future research should recruit a wider range of participants with regard to age. Finally, because of the limited geographic region and age range, our results may not be generalizable across other areas in the U.S.

### 4.6. Public Health Implications

The strong association between people who have taken PrEP and have ever taken PEP could be due to the fact that there is more knowledge and access for those who are already familiar with certain prophylaxis medications. If an at-risk person is seeing a primary care provider consistently to ensure they are taking the necessary precautions with PrEP, they could be more likely to also try PEP if they are ever in an emergency situation. On the other hand, the association between methamphetamine and PEP use could mean that participants who use methamphetamine are more likely to engage in unprotected sex or not use PrEP. This information can be used to increase public health campaigns targeting young SMM who use methamphetamine to increase primary and secondary HIV prevention efforts, specifically educating and providing community outreach about using biomedical prophylaxis therapies, while also encouraging safer sex. 

Recent interventions to reduce HIV transmission among methamphetamine-using SMM have focused on understanding psychosocial factors and social structures through cognitive behavioral therapy [32]. Further research is necessary to understand the underpinnings and motivations behind PEP uptake and the demographic and behavioral patterns associated with methamphetamine use—particularly in at-risk groups such as young SMM in NYC—in order to develop effective interventions.

## 5. Conclusions

The purpose of this study was to evaluate and understand what demographic and behavioral factors are associated with ever use of PEP among a young population of SMM, and particularly recent methamphetamine use in any form. Our findings suggest that methamphetamine use was a significant predictor for ever using PEP in an emerging population of young SSM at risk for HIV infection. This study highlights the critical need for increasing public health interventions among vulnerable and high-risk populations in the NYC area.

## Figures and Tables

**Table 1:** Characteristics of P18 Cohort Study participants from baseline Wave II sample (*n* = 665).

Variable	*n*	%
**Race/Ethnicity**	665	100
Hispanic/Latino	212	31.88%
Black Non-Hispanic	170	25.56%
Asian Non-Hispanic	49	7.37%
Mixed Non-Hispanic	43	6.47%
Native American/Other Non-Hispanic	23	3.46%
White Non-Hispanic	168	25.26%
**Born in the U.S.**	-	-
No	104	15.64%
Yes	560	84.21%
Missing	1	0.15%
**Sexual orientation**	-	-
Identifies as “not exclusively homosexual”	118	17.74%
Identifies as “exclusively homosexual”	547	82.26%
**Annual personal income**	**-**	**-**
Lower (<5 k)	219	32.93%
Middle (5 k–19,999)	200	30.08%
Upper (>20 K)	214	32.18%
Missing	32	4.81%
**Current school enrollment**	-	-
No	443	66.62%
Yes	222	33.38%
**Highest level of education completed**	-	-
High school diploma or less	318	47.82%
Associate degree	78	11.73%
Bachelor’s degree	260	39.10%
Graduate degree	8	1.20%
Missing	1	0.15%
**HIV Status**	-	-
HIV−	629	94.59%
HIV+	30	4.51%
Test not given	6	0.90%
**Methamphetamine Use (In the past 6 months)**	-	-
No	617	92.78%
Yes	43	6.47%
Missing	5	0.75%
**PrEP Use**	-	-
No	459	69.02%
Yes	59	8.87%
Missing	147	22.11%
**PEP Use**	-	-
No	439	66.02%
Yes	57	8.57%
Missing	169	25.41%

**Table 2:** PEP ever use by participant characteristics, PrEP ever use, and recent methamphetamine use (*n* = 429) ^a^.

	PEP Ever Use				
	No (*n* = 378)		Yes (*n* = 51)		
	*n*	%	*n*	%	*p*-Value (α = 0.05) ^b^
**Race/Ethnicity**					0.13 ^b^
Hispanic/Latino	125	90.58%	13	9.42%	
Black NH	76	80.85%	18	19.15%	
Asian NH	21	80.77%	5	19.23%	
Mixed NH	24	88.89%	3	11.11%	
Native American/Other NH	12	92.31%	1	7.69%	
White NH	120	91.60%	11	8.40%	
**Born in the U.S.**					0.54
No	53	91.38%	5	8.62%	
Yes	325	87.60%	46	12.40%	
**Sexual orientation**					0.83
Identifies as “not exclusively homosexual”	51	86.44%	8	13.56%	
Identifies as “exclusively homosexual”	327	88.38%	43	11.62%	
**Annual Personal Income**					0.18
Lower (<5 k)	130	92.20%	11	7.80%	
Middle (5 k–19,999)	105	85.37%	18	14.63%	
Upper (>20 K)	143	86.67%	22	13.33%	
**Current school enrollment**					0.36
No	251	89.32%	30	10.68%	
Yes	127	85.81%	21	14.19%	
**Highest level of education completed**					0.027 ^b^
High school diploma or less	162	85.26%	28	14.74%	
Associate degree	38	84.44%	7	15.56%	
Bachelor’s degree	174	92.55%	14	7.45%	
Graduate degree	4	66.67%	2	33.33%	
**HIV Status**					0.40 ^b^
HIV−	375	88.24%	50	11.76%	
HIV+	3	75.00%	1	25.00%	
**PrEP Ever Use**					<0.0001
No	352	92.63%	28	7.37%	
Yes	26	53.06%	23	46.94%	
**Methamphetamine Use (In the past 6 months)**					0.0015 ^b^
No	361	89.58%	42	10.42%	
Yes	17	65.38%	9	35.62%	

^a^ There were 236 participants with missing values removed from the analytic sample. ^b^ Fisher’s exact test was used for analysis instead of χ^2^.

**Table 3:** Binary logistic regression examining associations between ever PEP use and individual characteristics, PrEP use, and methamphetamine use.

Variable	OR (95% CI)	*p*-Value	AOR (95% CI)	*p*-Value
**Race/Ethnicity**				
White (Reference)	1		1	
Hispanic/Latino	1.13 (0.49–2.68)	0.77	1.01 (0.38–2.69)	0.99
Black NH	2.58 (1.17–5.93)	0.02	2.47 (0.94–6.74)	0.07
Asian NH	2.6 (0.76–7.96)	0.11	3.16 (0.75–12.04)	0.10
Mixed NH	1.43 (0.31–4.99)	0.61	1.27 (0.23–5.44)	0.76
Native American/Other NH	0.91 (0.05–5.33)	0.93	0.45 (0.02–3.44)	0.51
**Highest level of education completed**				
High school diploma or less (Reference)	1		1	
Associate degree	1.06 (0.40–2.49)	0.9	1.34 (0.44–3.63)	0.58
Bachelor’s degree	0.46 (0.23–0.90)	0.03	0.39 (0.17–0.88)	0.03
Graduate degree	2.88 (0.39–15.47)	0.24	1.93 (0.20–14.35)	0.53
**Methamphetamine Use**				
Yes	4.54 (1.83–10.62)	0.0007	6.07 (2.10–16.86)	0.0006
**PrEP Ever Use**				
Yes	11.09 (5.63–22.06)	<0.0001	16.0 (7.41–35.95)	<0.001

CI = Confidence Interval. OR = Odds Ratios. AOR = Adjusted Odds Ratios.

## Data Availability

To protect the confidentiality of P18 cohort participants, we do not make data freely available. Data are housed at the Rutgers School of Public Health’s Center for Health, Identity, Behavior and Prevention Studies (CHIBPS), where the P18 cohort PI (P.H.) is housed.

## Appendix A

**Table A1:** Unprotected Sexual Behavior by PEP Use.

	PEP Use Ever-No		PEP Use Ever-Yes		
	Mean	Standard Deviation	Mean	Standard Deviation	*p*-Value
Total unprotected oral given	3.43	4.39	4.14	4.45	0.26
Total unprotected oral received	3.51	4.27	4.35	4.28	0.16
Total unprotected anal insertive	0.87	2.65	1.37	2.75	0.20
Total unprotected anal receptive	1.05	2.49	1.23	2.54	0.62
Total unprotected vaginal insertive	0.01	0.16	0.00	0.00	0.13
Total unprotected mutual masturbation	2.31	3.31	3.46	5.82	0.15
Total unprotected rimming received	1.27	2.74	1.56	3.12	0.50
Total unprotected rimming given	1.06	2.72	1.40	3.05	0.42
Total unprotected fingering received	1.07	2.66	0.91	2.08	0.60
Total unprotected fingering given	0.79	2.22	1.44	2.84	0.10
Unprotected fisting received	0.01	0.15	0.02	0.13	0.66
Unprotected fisting insertive	0.01	0.18	0.05	0.29	0.33
Total Unique partners	2.48	2.76	3.33	3.67	0.09
